# The effect of data balancing approaches on the prediction of metabolic syndrome using non-invasive parameters based on random forest

**DOI:** 10.1186/s12859-024-05633-9

**Published:** 2024-01-11

**Authors:** Sahar Mohseni-Takalloo, Hadis Mohseni, Hassan Mozaffari-Khosravi, Masoud Mirzaei, Mahdieh Hosseinzadeh

**Affiliations:** 1 School of Public Health, Bam University of Medical Sciences, Bam, Iran; 2 Research Center for Food Hygiene and Safety, School of Public Health, Shahid Sadoughi University of Medical Sciences, Yazd, Iran; 3 Present Address: Department of Nutrition, School of Public Health, Shahid Sadoughi University of Medical Sciences, Yazd, Iran; 4 Computer Engineering Department, Shahid Bahonar University of Kerman, Kerman, Iran; 5 Yazd Cardiovascular Research Centre, Non-Communicable Diseases Research Institute, Shahid Sadoughi University of Medical Sciences, Yazd, Iran

**Keywords:** Metabolic syndrome, Machine learning, Random forest, Data balancing, SMOTE, SplitBal

## Abstract

**Background:**

Metabolic syndrome (MetS) is a cluster of metabolic abnormalities (including obesity, insulin resistance, hypertension, and dyslipidemia), which can be used to identify at-risk populations for diabetes and cardiovascular diseases, the main causes of morbidity and mortality worldwide. The achievement of a simple approach for diagnosing MetS without needing biochemical tests is so valuable. The present study aimed to predict MetS using non-invasive features based on a successful random forest learning algorithm. Also, to deal with the problem of data imbalance that naturally exists in this type of data, the effect of two different data balancing approaches, including the Synthetic Minority Over-sampling Technique (SMOTE) and Random Splitting data balancing (SplitBal), on model performance is investigated.

**Results:**

The most important determinant for MetS prediction was waist circumference. Applying a random forest learning algorithm to imbalanced data, the trained models reach 86.9% and 79.4% accuracies and 37.1% and 38.2% sensitivities in men and women, respectively. However, by applying the SplitBal data balancing technique, the best results were obtained, and despite that the accuracy of the trained models decreased by 7.8% and 11.3%, but their sensitivity improved significantly to 82.3% and 73.7% in men and women, respectively.

**Conclusions:**

The random forest learning method, along with data balancing techniques, especially SplitBal, could create MetS prediction models with promising results that can be applied as a useful prognostic tool in health screening programs.

## Background

Metabolic syndrome (MetS), a public health problem worldwide, is a condition associated with multiple metabolic abnormalities (including obesity, hyperglycemia, hypertension, and dyslipidemia), which can be used to identify at-risk populations for numerous non-communicable diseases, including cardiovascular diseases, type 2 diabetes, and stroke [1]. The economic burden of healthcare, social costs, and lost productivity associated with these diseases are trillions of dollars per year [2]. Therefore, it is very valuable to achieve a simple and effective approach to diagnosing MetS using non-invasive features without requiring biochemical tests [3].

One of the prominent topics in public health and preventive medicine is to predict diseases, such as MetS, with acceptable accuracy using existing datasets and to perform appropriate interventions [4]. Compared to classical approaches (e.g., logistic regression), some recent machine learning algorithms have better performance in MetS prediction [5]. In fact, these algorithms consider the nonlinearity and complex relationships between multiple risk factors and discover unknown patterns, making the diagnostic process more objective and reliable [6]. One of the machine learning methods that yielded promising results in disease prediction is the Random Forest (RF) algorithm, a method that develops multiple decision trees (predictors) based on a different combination of data features and shows the importance of these features used in its predictions [5]. It has been shown that random forest is one of the best machine-learning methods for predicting metabolic syndrome [3, 5, 7–10]. Also, the random forest has performed better than traditional models such as logistic regression in predicting metabolic syndrome [5, 8]. The accuracies of MetS prediction based on random forest using non-invasive features in two previous studies were 78.8% and 83.8%, respectively, depending on the population characteristics and input features [5, 11].

However, one of the main challenges that affect the performance of machine learning methods (including random forest) is data imbalance, a problem commonly found in medical science data [12]. Generally, in a population, the number of healthy people is greater than the number of patients, which usually encourages or biases the learning process to be done mostly based on the larger class, i.e., healthy subjects, while the smaller class might be ignored. Consequently, the learned model might have promising performance in classifying healthy subjects, but its performance is not acceptable in diagnosing disease in patients [12], which has higher importance. To deal with this common challenge, data balancing approaches are used as over-sampling or under-sampling techniques. Although the purpose of both approaches is to balance the data so that different classes contain a similar amount of data, they use different points of view. Over-sampling is the process that increases the number of data samples in the minority class either randomly through the repetition of existing data or by generating new samples. On the other side, under-sampling removes some samples from the majority class randomly or divides the majority class into several smaller ones based on different criteria [13]. In this regard, previous studies have shown that using over-sampling approaches can improve the performance of machine learning models in predicting MetS [5, 14].

Due to the high prevalence of MetS in our population (about 30%) [15], the present study aimed to (1) predict MetS using non-invasive features based on the random forest models and (2) investigate the effects of both over-sampling and under-sampling on the prediction capabilities of the learned models, as each one has its own advantages and disadvantages.

## Methods

### Study design and population

The present cross-sectional study was performed under the framework of the baseline survey of two population-based cohort studies, the Shahedieh Cohort Study and the Yazd Health Study (YaHS), which included 19,933 adults from the Yazd Greater Area located in the central part of Iran. Detailed information about the design and population of these studies was published elsewhere [16, 17]. In brief, the YaHS study recruited 9962 people aged 20–70 years from the urban areas of Yazd in 2014–2015, and biochemical assessment was done only in 3,748 persons who gave consent. Also, the Shahedieh study included 9971 adults aged 35–70 years from Yazd Greater Area, including Shahedieh, Zarch, and Ashkezar in 2016. In the current study, participants were excluded if they met any of the following criteria: (1) incomplete data on demographic, anthropometric, biochemical, blood pressure, physical activity, family history of diseases, smoking, or drug use; (2) had a history of cardiovascular disease, diabetes, hypertension, fatty liver disease, thyroid disease, or different types of cancer; (3) body mass index (BMI) < 18.5 kg/m^2^ or BMI > 40 kg/m^2^; and (4) pregnancy or lactation. Finally, 5910 individuals (1314 from YaHS and 4596 from Shahedieh) were eligible for inclusion in our analysis. Informed consent was obtained from all participants, and the present study has been approved by the ethics committee of Shahid Sadoughi University of Medical Sciences (approval code: IR.SSU.SPH.REC.1399.202).

### Clinical and biomedical assessment

All anthropometric indices were measured by trained investigators. Weight was recorded with minimum clothing using a portable digital scale (Omron BF511, Nagoya, Japan) with an accuracy of 0.1 kg. Height was measured in the standing position without shoes while their heads, shoulders, buttocks, and heels were touching the wall, using a non-stretchable tape meter to the nearest centimeter. Waist circumference and hip circumference were measured in the standing position by non-stretch tape placed midway between the iliac crest and the lowest rib and over the largest part of the buttocks, respectively, with an accuracy of 0.5 cm. BMI was obtained by dividing weight in kilograms by the square of height in meters. Systolic and diastolic blood pressures were measured in the sitting position three times at 5-min intervals using Reichter electronic sphygmomanometers (Model N-Champion, Reister GMBH, Germany), which were calibrated regularly. The mean of the measurements was recorded as the individual's blood pressure. Participants were asked to fast over the night (for 8 to 12 h), and then blood samples were collected from each enrollee. Glucose and lipid concentration measurements were performed according to a standard laboratory protocol using Pars Azmoon kits and calibrated auto-analyzers [16].

### Other variables assessment

The demographic and medical history data were collected by applying a validated questionnaire containing age, gender, physical activity level, smoking status, education level, drug consumption, and family history of chronic diseases. Moreover, the Iranian version of the International Physical Activity Questionnaire (IPAQ) was used to obtain individual physical activity data (type, frequency, and time of each exercise) [18]. Finally, physical activity was reported on a metabolic equivalent per week (MET-h/wk) basis [19].

### Diagnosis of metabolic syndrome

According to the National Cholesterol Education Program Adult Treatment Panel III (ATP III) guidelines [20], having at least three of the following criteria shows the presence of MetS: (1) waist circumference (WC) > 88 cm in women and > 102 cm in men; (2) fasting blood glucose (FBG) ≥ 100 mg/dl; (3) serum high-density lipoprotein cholesterol (HDL-C) < 50 mg/dl in women and < 40 mg/dl in men; (4) serum triglycerides (TG) ≥ 150 mg/dl; and (5) systolic blood pressure (SBP) ≥ 130 mmHg and/or diastolic blood pressure (DBP) ≥ 85 mmHg.

### Dataset creation

According to the previous literature, 20 potential MetS predictors and outcome variables were extracted from the Shahedieh Cohort Study and Yazd Health Study datasets, including clinical and biomedical data (weight, height, BMI, WC, hip circumference (HC), waist-to-height ratio (WHiR), waist-to-hip ratio (WHR), SBP, DBP, TG, HDL-C, FBG), and demographic and medical history data (sex, age, physical activity, education status, smoking status, drug consumption, family history in first-degree relatives for diabetes and cardiovascular disease).

### Data preprocessing

**Fig. 1:**
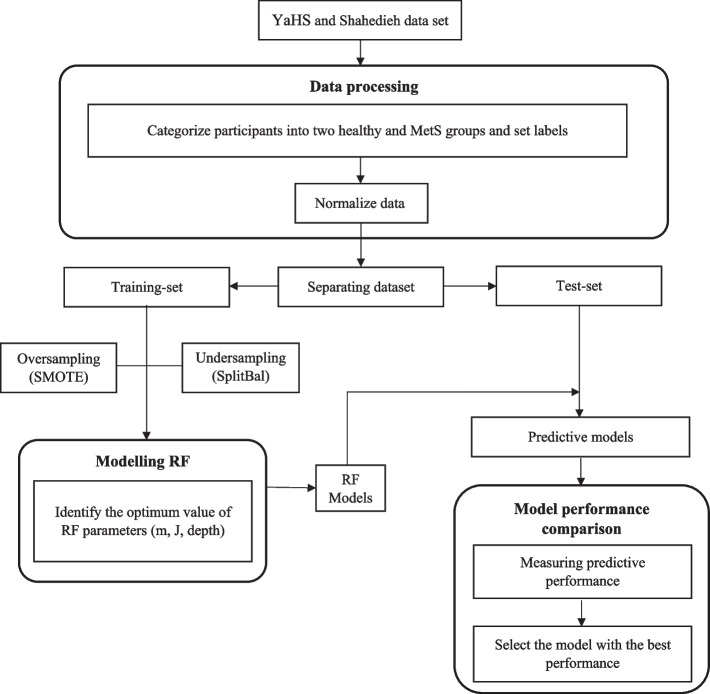
Flowchart of data processing for metabolic syndrome classification with RF. RF: Random Forest; SMOTE: Synthetic Minority Oversampling Technique; SplitBal: Random Splitting data balancing

Figure 1 illustrates the steps of this research. After gathering the dataset, considering that the ranges of features' values were different, which can affect the learning process in learning models, the "standard scaler" normalization method was used to make sure that the values of all features were in the [−1, 1] interval [21].

As mentioned before, another factor that promisingly affects the performance of prediction models is data balancing. According to the data imbalance between the two available classes (MetS and healthy classes) in the present study and being aware of the negative effects of imbalanced data on the learning process, it is a good idea to investigate how data balancing methods may influence the learning process.

To have a fair judgment, one has to consider both over-sampling and under-sampling viewpoints when balancing the data. In the present study, we have used the famous Synthetic Minority Over-sampling Technique (SMOTE) method [22] from the over-sampling viewpoint and the Random Splitting data balancing (SplitBal) method [23] from the under-sampling viewpoint. The SMOTE aims to extend the minority class by generating synthetic data samples using the neighborhood principle approach. It generates new samples based on combining available neighbor samples, so each generated data sample lies among its neighbors and has similar characteristics to them. In the SplitBal method, the imbalanced dataset is balanced by randomly dividing the majority class data into N groups, so that the number of samples in each group is almost the same as the number of samples in the minority class. Then, the samples of each group from the majority class are merged with the samples of the minority class to create N balanced sub-datasets, which are used to train N separate learning models. Therefore, N separate models are trained, and their results are combined using an ensemble technique.

In summary, after normalizing the data using “standard scalar” the data is divided into a training set and a test set. Then, data balancing is done on the training set by the SMOTE or SplitBal method before training the model. However, the performance of each trained model is investigated on the test set and the unseen data during the training process.

### Random forest method

Random forest is a supervised machine learning method that is widely used in disease prediction. In order to achieve high accuracy, this ensemble-based technique integrates multiple decision trees based on the bagging method. Each tree is designed using randomly selected features from all data features and defines classification as the vote. Finally, the data class is determined based on the most votes among all the trees in the forest. In the present research, the most proper values for random forest parameters (including m, the number of features chosen to create each decision tree, and J, the number of decision trees to be used in the forest) were determined using a grid search. In addition, by using a random forest, the importance of each variable in data classification is determined [24].

### Evaluation of model performance

As mentioned before, each trained model should be evaluated on data unseen in training. The present research has applied a fivefold cross-validation method (splitting the dataset into 5 equal subsets: 4 subsets are considered the training set, and 1 subset forms the test set). Also, to increase the results' reliability, cross-validation was repeated five times and the mean of all the results was reported as the performance of the model.

The performance of the models was measured using the following criteria: accuracy (ACC), sensitivity (Sen), specificity (Spe), Positive Prediction Value (PPV), Negative Prediction Value (NPV), F1-score, and receiver operating characteristic (ROC) curve. Considering the high importance of sensitivity over specificity in the timely diagnosis and treatment of disease, as well as the ultimate purpose of these models (which was to screen people with MetS), parameter tuning was done such that selected models had the highest sensitivity while maintaining acceptable accuracy. It is worth mentioning that the performance of the MetS prediction models was assessed before and after data balancing techniques.

### Statistical analysis

All machine learning analyses were performed using the Python software package incorporated in the ANACONDA NAVIGATOR (version 1.00). Also, the SPSS statistical package, version 22.0, was applied for statistical analyses. Two independent t-tests and chi-square were used to compare the two studied groups (the MetS group and the healthy group). A *P*-value < 0.05 was considered statistically significant.

## Results

### Characteristics of the study population

A total of 5910 participants (58.6% men and 41.4% women) were included in the study, and 18.8% of men and 34.0% of women were classified as MetS according to ATP III criteria. In both sexes, the mean age of the healthy group was significantly lower than the MetS group (age: 48.6 ± 10.5 vs 51.1 ± 10.3 years in men and 46.1 ± 10.2 vs 49.7 ± 9.8 years in women; *P* < 0.001). In addition, by increasing educational level, the prevalence of MetS decreased (*P* = 0.001 in men and *P* < 0.001 in women). Also, no significant difference was observed in the current smoking status, use of drugs, and history of diabetes and cardiovascular diseases in the first-degree relatives between the two groups. The participant characteristics are detailed in Table 1.

**Table 1:** Characteristics of participants of Yahs (2014–2015) and Shahedieh (2015–2016) recruitment phase Yazd Greater Area

Age		Men			Women			Total		
		MetS group (n = 548)	Healthy group (n = 2918)	*P* value	MetS group (n = 620)	Healthy group (n = 1824)	*P* value	MetS group (n = 1168)	Healthy group (n = 4742)	*P* value*
		51.1 ± 10.3**	48.6 ± 10.5	< 0.001	49.7 ± 9.8	46.1 ± 10.2	< 0.001	50.4 ± 10.0	47.6 ± 10.4	< 0.001
BMI		29.7 ± 3.7	26.1 ± 3.7	< 0.001	30.4 ± 4.0	27.5 ± 4.4	< 0.001	30.1 ± 3.9	26.6 ± 4.0	< 0.001
WC		102.2 ± 10.0	91.5 ± 9.8	< 0.001	99.9 ± 8.8	91.3 ± 11.1	< 0.001	101.0 ± 9.4	91.4 ± 10.3	< 0.001
SBP (mmHg)		123.8 ± 17.1	110.0 ± 14.0	< 0.001	117.0 ± 19.2	104.1 ± 14.0	< 0.001	120.2 ± 18.5	107.7 ± 14.3	< 0.001
DBP (mmHg)		78.4 ± 12.2	69.3 ± 10.3	< 0.001	73.1 ± 12.8	65.5 ± 10.3	< 0.001	75.6 ± 12.8	67.8 ± 10.5	< 0.001
FBS (mg/dl)		111.8 ± 37.5	93.5 ± 17.0	< 0.001	104.5 ± 25.1	91.7 ± 14.8	< 0.001	107.9 ± 31.7	92.8 ± 16.2	< 0.001
TG (mg/dl)		263.1 ± 142.8	155.3 ± 87.0	< 0.001	204.6 ± 98.9	114.0 ± 52.1	< 0.001	232.1 ± 124.9	139.4 ± 78.1	< 0.001
Cholestrol (mg/dl)		201.1 ± 45.1	188.0 ± 38.3	< 0.001	202.3 ± 39.4	188.0 ± 40.5	< 0.001	201.7 ± 42.2	188.0 ± 39.2	< 0.001
HDL-C (mg/dl)		41.8 ± 10.2	49.6 ± 10.1	< 0.001	47.4 ± 10.3	58.4 ± 11.3	< 0.001	44.8 ± 10.6	53.0 ± 11.4	< 0.001
Physical activity (MET-h/wk)		34.5 ± 15.6	39.1 ± 14.0	< 0.001	29.8 ± 15.3	34.3 ± 13.8	< 0.001	32.0 ± 15.6	37.2 ± 14.1	< 0.001
	Illiterate	71 (13.0)	233 (8.0)	0.001	158 (25.5)	261 (14.3)	< 0.001	229 (19.6)	494 (10.4)	< 0.001
	Elementary and Middle school	240 (43.8)	1211 (41.5)		298 (48.1)	855 (46.9)		538 (46.1)	2066 (43.6)	
	High school, and associate	158 (28.8)	963 (33.0)		116 (18.7)	485 (26.6)		274 (23.5)	1448 (30.5)	
	Bachelor’s	64 (11.7)	411 (14.1)		43 (6.9)	195 (10.7)		107 (9.2)	606 (12.8)	
	Master’s and PhD	15 (2.7)	102 (3.5)		4 (0.6)	27 (1.5)		19 (1.6)	129 (2.7)	
Current Smoking, n (%)	Yes (daily)	102 (18.6)	601 (20.6)	0.31	3 (0.5)	7 (0.4)	0.57	105 (9.0)	608 (12.8)	< 0.001
	Yes (sometimes)	27 (4.9)	174 (6.0)		0 (0.0)	3 (0.2)		27 (2.3)	177 (3.7)	
	No	419 (76.5)	2142 (73.4)		617 (99.5)	1815 (99.5)		1036 (88.7)	3957 (83.4)	
Use drugs, n (%)		128 (23.4)	762 (26.1)	0.20	4 (0.6)	11 (0.6)	1.00	132 (11.3)	773 (16.3)	< 0.001
Diabetes FH-1, n (%)		271 (49.5)	1386 (47.5)	0.40	317 (51.1)	908 (49.8)	0.64	588 (50.3)	2294 (48.4)	0.25
Cardiac Disease FH-1, n (%)		198 (36.1)	1080 (37.0)	0.74	239 (38.5)	719 (39.4)	0.74	437 (37.4)	1799 (37.9)	0.76

MetS: Metabolic Syndrom; WC: Waist Circumference; BMI: Body Mass Index; FH-1: Family history in first-degree relatives; MI: Myocardial Infarction; SBP: Systolic Blood Pressure; DBP: Diastolic Blood Pressure; FBG: Fasting Blood Glucose; TG: Tryglicerid; HDL-C: High-Density Lipoprotein Cholesterol

*Two independent t-tests was used for quantitative variables, and chi-square was used for qualitative variables. ** mean ± SD

The obtained results from training random forest models on this population are presented here from different perspectives. First, we show how different data balancing methods affect the performance of learned models in predicting MetS. Then, we investigate whether they influence the importance of different features in the learned models. This way, one can infer how learning methods are affected by imbalanced data and if data balancing can improve the performance of these methods or change the importance of different features used by these methods.

### Comparison of random forest algorithms according to different data balancing methods

To investigate the effects of data balancing methods, the performance of different trained models is demonstrated in Table 2. When MetS was predicted using imbalanced data based on random forest, although the accuracy was high in men, women, and the whole population (86.9%, 79.4%, and 83.6%, respectively), the sensitivity, i.e., the ability to distinguish patients, of the models was low (37.1%, 38.2%, and 35.5%, respectively). Using the SMOTE method, the accuracy of the models decreased a little (79.1%, 67.6%, and 72.1% in men, women, and the whole population, respectively), but their sensitivity improved significantly (78.1%, 73.4%, and 77.6% in men, women, and the whole population, respectively). Applying the SplitBal technique slightly improved the performance of the models compared to the SMOTE, so the sensitivity increased by 3.8% in men and 0.3% in women.

**Table 2:** Models performance in prediction of metabolic syndrome based on non-invasive features

Data status		Accuracy	Sensitivity	Specificity	PPV	NPV	f1-Score	ROC	Model type & parameters
Imbalance data	Men	86.9	37.1	96.2	65.2	89.1	47.2	0.86	J = 200, m = 6
	Women	79.4	38.2	93.4	66.7	81.7	48.3	0.79	J = 200, m = 5
	total	83.6	35.9	95.3	65.3	85.8	46.3	0.83	J = 200, m = 6
Balancing data based on SMOTE	men	79.1	78.1	79.2	41.5	95.1	54.2	0.86	J = 200, m = 4
	women	67.6	73.4	65.7	42.2	88.0	53.5	0.79	J = 200, m = 4
	total	72.1	77.6	70.7	39.5	92.8	52.3	0.83	J = 200, m = 4
Balancing data based on SplitBal	men	79.1	82.3	78.3	41.7	96.0	55.3	0.86	J = 200, m = 5
	women	68.1	73.7	66.1	42.6	88.1	54.0	0.80	J = 200, m = 4
	total	74.2	76.3	73.6	41.7	92.7	53.9	0.83	J = 200, m = 4

PPV: Positive Prediction value; NPV: Negative Prediction value; m: the number of variables to create each decision tree; J: the number of decision trees to be used in the forest

**Fig. 2:**
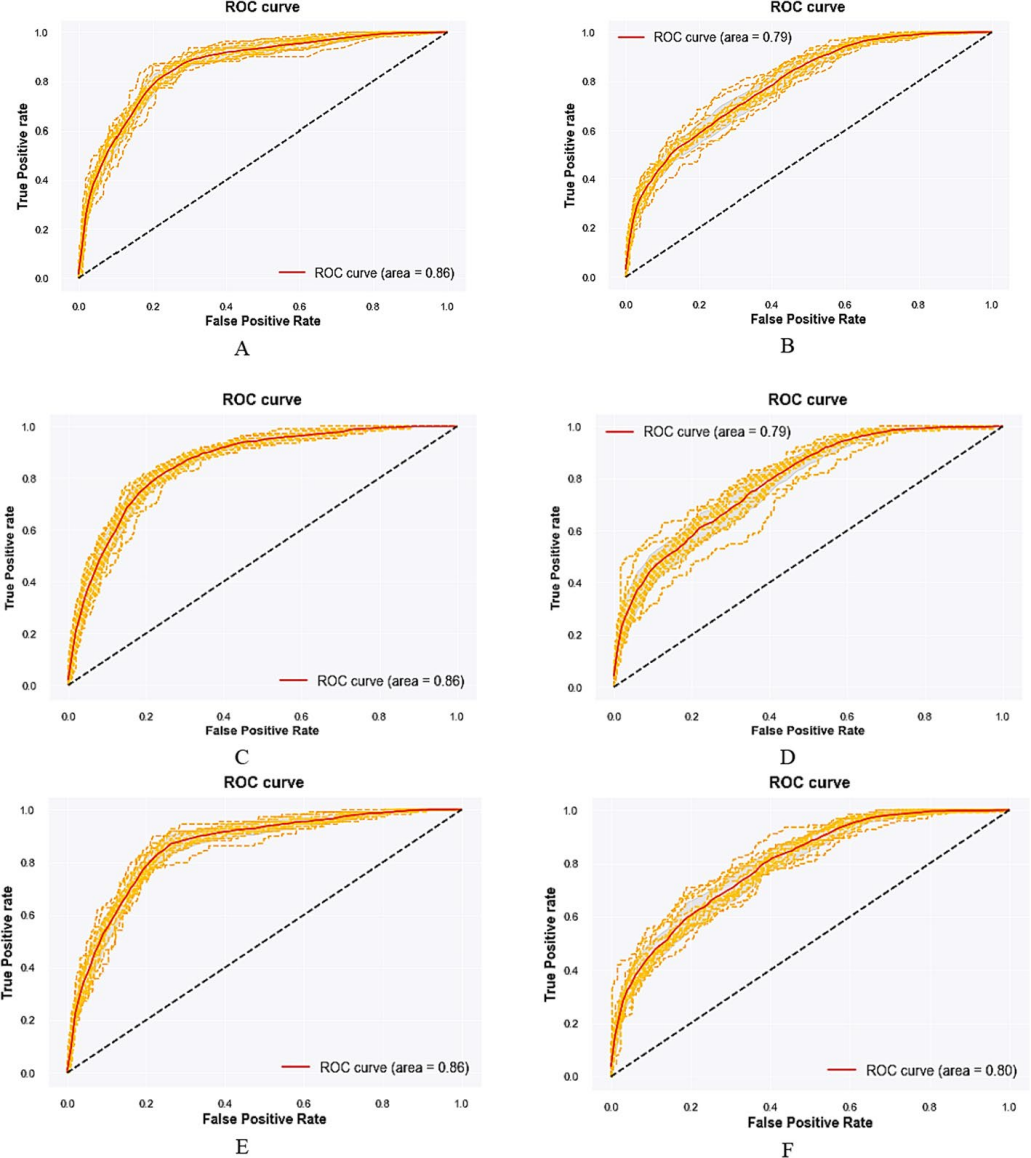
MetS prediction ROC curves based on different data balancing methods by sex. **A** ROC curve in men using imbalance data; **B** ROC curve in women using imbalance data; **C** ROC curve in men using the SMOTE method; **D** ROC curve in women using the SMOTE method; **E** ROC curve in men using the SplitBal method; **F** ROC curve in women using the SplitBal method

Figure 2 displays the models' performance based on the ROC curve. The ROC curve illustrates the true positive rate against the false positive rate at various threshold settings to indicate the predictive ability of a binary classifier system. The average area under the curve was almost equal before and after using data balancing methods (0.86 in men and 0.79 in women).

### Comparison of variables' importance in the unbalanced and balanced datasets

**Fig. 3:**
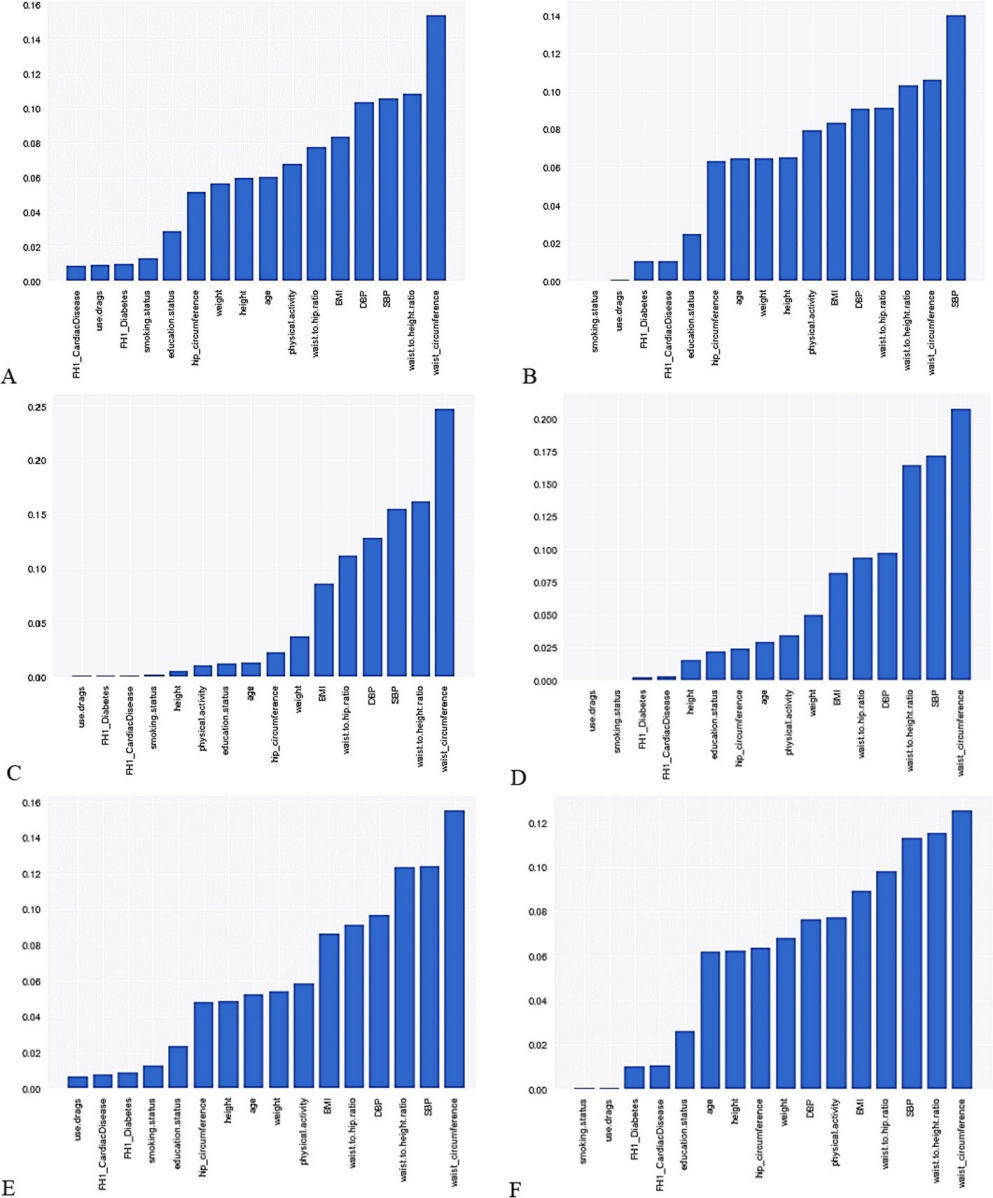
Feature importance in the MetS prediction model based on different data balancing methods. **A** Features importance on imbalanced data in men; **B** features importance on imbalanced data in women; **C** features importance based on the SMOTE method in men; **D** features importance based on the SMOTE method in women; **E** features importance based on the SplitBal method in men; **F** features importance based on the SplitBal method in women. BMI: body mass index, SBP: systolic blood pressure, DBP: diastolic blood pressure, FH1: family history in a first-degree relative

The importance of variables with and without data balancing techniques by sex is shown in Fig. 3. In all models, there were six determining features for predicting MetS (including WC, WHiR, WHR, SBP, DBP, and BMI). The considerable issue was that, by using the SMOTE technique, the discriminating power of models in determining important features was improved.

## Discussion

The results of the present study indicated that the performance of the models on imbalanced data was indefensible, and despite the appropriate accuracy of the designed models, their sensitivity was low (accuracies were 86.9% and 79.4% and sensitivities were 37.1% and 38.2% in men and women, respectively). Moreover, the outcomes confirmed that by applying data balancing techniques, especially SplitBal, the models’ performance improved, and despite some decrease in accuracy, sensitivity increased significantly (accuracies were 79.1% and 68.1% and sensitivities were 82.3% and 73.7% in men and women, respectively). These obtained results imply that, generally, data balancing methods can enhance the learning efficiency of the models by preventing them from being biased toward a specific (or majority) class. As it is obvious from Table 2, the specificity is high in the model trained on imbalanced data, which means the model is very good at classifying healthy people (the majority class), while sensitivity is very low, and patients with MetS cannot be recognized successfully. However, this is not the issue in models trained using balanced data, which proves the positive impact of data balancing on the learning process.

As the prevalence of MetS has increased worldwide in recent decades, it has become a serious public health problem [25]. Therefore, the early diagnostic prediction of MetS without requiring biochemical tests can be considered a health priority. Recently, machine learning algorithms have made considerable progress in predicting and diagnosing diseases by discovering unknown patterns and relationships [26]. The present study applied machine learning technology (random forest) to construct MetS prediction models using non-invasive variables in Iranian adults and evaluated the effect of data balancing techniques on the performance of the models. Due to sex-related differences in the prevalence of MetS and its risk factors, the analysis was performed on men and women separately [27]. Previous studies have shown that among machine learning methods, the random forest has one of the best performances in predicting MetS [3, 5, 7–10]. Also, in comparison with traditional logistic regression approaches, the random forest can predict MetS with a 3% increase in accuracy and sensitivity [5]. In another study, random forest predicted MetS with 2% and 7% higher accuracy and sensitivity, respectively, than logistic regression [8]. The random forest method has three important advantages: (1) a large number of decision trees are generated by randomly selecting samples and features. For each new sample, each of the decision trees determines the class of the sample (here it has MetS or not), and finally, by voting from all the trees, the final result of the random forest prediction is determined. (2) Those samples that are not used to generate forest trees can be used to determine the performance of the model, which reduces the prediction error. (3) This method can provide useful insight into the features’ importance, which can be applied to model interpretations [28]. Therefore, in the present study, the random forest method was used to investigate the effect of different data-balancing techniques on the prediction of metabolic syndrome.

In fact, one of the challenges of using machine learning methods in the field of medical sciences is data imbalance, which can affect the performance of learned models [12]. Over-sampling and under-sampling data balancing approaches (e.g., SMOTE and SplitBal) can help to cope with this challenge [29]. Previously, other researchers have used machine learning methods to predict MetS. Kim et al. indicated that by using the SMOTE balancing technique, the performance of models designed with nine machine learning methods, including random forest, improved, so that a significant increase in accuracy (from 77 to 81%) and sensitivity (from 62 to 83%) was observed in the random forest model based on demographic and anthropometric data [5]. Another study in Mexico using data balancing and random forest algorithms was able to achieve acceptable results in predicting MetS (accuracy and sensitivity were 85% and 95%, respectively). In this study, biochemical data was used in addition to anthropometric, lifestyle, and blood pressure data [7]. Also, Vrbaski et al. predicted MetS with a sensitivity of 91% and a specificity of 94% using the random forest method based on low-cost and non-invasive variables (sex, age, body mass index, waist-to-height ratio, systolic and diastolic blood pressures) in an almost balanced dataset [3]. On the other hand, in the Park et al. study, which did not use data balancing techniques, the performance of six machine learning methods, including a random forest, in predicting MetS (based on age, sex, education level, marital status, body mass index, stress, physical activity, alcohol consumption, and smoking variables) was not defensible (accuracy 78% and sensitivity 36%) [11]. Previously, the Choe et al. study also showed that the performance of predictive models of MetS (based on non-invasive clinical data) that were designed using imbalanced data was not acceptable (accuracy of 78% and sensitivity of 8% in the random forest model) [30].

In the present study, the most important features in MetS prediction were WC, WHiR, WHR, SBP, DBP, and BMI in all models in both sexes. These results are aligned with other studies that have stated BMI, WC, WHiR, WHR, sex, and age as important non-invasive features in predicting MetS [5, 7, 10, 11]. The difference in the results of various studies can be related to the balance of the data, the studied population, and the input features used (including demographic, clinical, biochemical, blood pressure, genetic data, etc.). One noteworthy point in the present study findings was that, although the most important predictive features of MetS were the same in all models, the SMOTE technique showed more power in differentiating the importance of the features entered into the model. This finding is very valuable when the importance of features in prediction is closer to each other.

Several strengths can be considered for the present study. First, the effect of various data balancing methods on the performance of predictive models was investigated. Second, the importance of non-invasive risk factors was ranked, considering the impact of each factor on the prediction of MetS. Third, due to easy access to non-invasive parameters, the designed models could be a useful prognostic tool in routine clinical practice by physicians and also in personal health applications. In addition, these models can be applied in public health screening programs for early diagnosis of MetS, followed by laboratory follow-up for a definitive diagnosis. Encouraging people with MetS to make lifestyle changes can significantly reduce metabolic risk factors and prevent many chronic diseases such as diabetes, cancer, and cardiovascular disease in the future. However, our study also has some limitations. The cross-sectional framework of the study does not allow us to find causal relationships between MetS and its risk factors. Also, our data were obtained solely from Yazd province in Iran; therefore, the generalizability of the results is limited. Adapting and validating these models for different Iranian populations is necessary.

## Conclusion

The present study indicated that random forest algorithms, using non-invasive features, could predict MetS defensively when applying data balancing methods, especially SplitBal (accuracies were 79.1% and 68.1% and sensitivities were 82.3% and 73.7% in men and women, respectively) in Iranian adults. These models can be applied for early diagnosis of MetS in everyday clinical practice, personal health applications, and public health screening programs. Also, the investigation of data balancing methods and their influence on the learning process can be used to train other successful diagnosis systems.

## Data Availability

The datasets analyzed during the current study are not publicly available but are available from the corresponding author upon reasonable request.

## Abbreviations

ACC: Accuracy
BMI: Body Mass Index
DBP: Diastolic blood pressure
FBG: Fasting blood glucose
HDL-C: High-density lipoprotein cholesterol
IPAQ: International Physical Activity Questionnaire
MET: Metabolic equivalent
MetS: Metabolic syndrome
NPV: Negative Prediction Value
SBP: Systolic blood pressure
Sen: Sensitivity
SMOTE: Synthetic Minority Over-sampling Technique
Spe: Specificity
SplitBal: Random Splitting data balancing
PPV: Positive Prediction Value
RF: Random forest
ROC: Receiver operating characteristic
TG: Triglycerides
WC: Waist circumference
WHiR: Waist-to-height ratio
WHR: Waist-to-hip ratio
YaHS: Yazd Health Study

## References

[1] Saklayen MG (2018). The global epidemic of the metabolic syndrome. Curr Hypertens Rep.

[2] Ricci G, Pirillo I, Tomassoni D, Sirignano A, Grappasonni I (2017). Metabolic syndrome, hypertension, and nervous system injury: epidemiological correlates. Clin Exp Hypertens.

[3] Vrbaski D, Vrbaski M, Kupusinac A, Ivanovic D, Stokic E, Ivetic D, Doroslovacki K (2019). Methods for algorithmic diagnosis of metabolic syndrome. Artif Intell Med.

[4] Dolley S. Big data’s role in precision public health. Front Public Health. 2018:68. 10.3389/fpubh.2018.00068 PMC5859342 29594091

[5] Kim J, Mun S, Lee S, Jeong K, Baek Y (2022). Prediction of metabolic and pre-metabolic syndromes using machine learning models with anthropometric, lifestyle, and biochemical factors from a middle-aged population in Korea. BMC Public Health.

[6] Dean J, Patterson D, Young C (2018). A new golden age in computer architecture: empowering the machine-learning revolution. IEEE Micro.

[7] Gutiérrez-Esparza GO, Infante Vázquez O, Vallejo M, Hernández-Torruco J (2020). Prediction of metabolic syndrome in a Mexican population applying machine learning algorithms. Symmetry.

[8] Datta S, Schraplau A, Da Cruz HF, Sachs JP, Mayer F, Böttinger E: A machine learning approach for non-invasive diagnosis of metabolic syndrome. In: 2019 IEEE 19th international conference on bioinformatics and bioengineering (BIBE). IEEE; 2019. p. 933–940.

[9] Xia S-J, Gao B-Z, Wang S-H, Guttery DS, Li C-D, Zhang Y-D (2021). Modeling of diagnosis for metabolic syndrome by integrating symptoms into physiochemical indexes. Biomed Pharmacother.

[10] Shimoda A, Ichikawa D, Oyama H (2018). Prediction models to identify individuals at risk of metabolic syndrome who are unlikely to participate in a health intervention program. Int J Med Inform.

[11] Park J-E, Mun S, Lee S (2021). Metabolic syndrome prediction models using machine learning and Sasang constitution type. Evid Based Complement Alternat Med.

[12] Junsomboon N, Phienthrakul T: Combining over-sampling and under-sampling techniques for imbalance dataset. In: Proceedings of the 9th international conference on machine learning and computing; 2017. p. 243–247.

[13] Shamsudin H, Yusof UK, Jayalakshmi A, Khalid MNA: Combining oversampling and undersampling techniques for imbalanced classification: a comparative study using credit card fraudulent transaction dataset. In: 2020 IEEE 16th International Conference on Control & Automation (ICCA). IEEE; 2020. p. 803–808.

[14] Karimi-Alavijeh F, Jalili S, Sadeghi M (2016). Predicting metabolic syndrome using decision tree and support vector machine methods. ARYA Atherosclerosis.

[15] Gholami S, Hazar N, Bagheri-Fahraji B, Azizi R, Ghadiri-Anari A, Nadjarzadeh A, Yaser Ghelmani S, Mirzaei M, Khayyatzadeh SS (2022). The association between metabolic syndrome and the consumption of some supplements. JNFS.

[16] Mirzaei M, Salehi-Abargouei A, Mirzaei M, Mohsenpour MA (2018). Cohort Profile: The Yazd Health Study (YaHS): a population-based study of adults aged 20–70 years (study design and baseline population data). Int J Epidemiol.

[17] Poustchi H, Eghtesad S, Kamangar F, Etemadi A, Keshtkar A-A, Hekmatdoost A, Mohammadi Z, Mahmoudi Z, Shayanrad A, Roozafzai F (2018). Prospective epidemiological research studies in Iran (the PERSIAN Cohort Study): rationale, objectives, and design. Am J Epidemiol.

[18] Moghaddam MB, Aghdam FB, Jafarabadi MA, Allahverdipour H, Nikookheslat SD, Safarpour S (2012). The Iranian Version of International Physical Activity Questionnaire (IPAQ) in Iran: content and construct validity, factor structure, internal consistency and stability. Sci World J.

[19] Ainsworth BE, Haskell WL, Whitt MC, Irwin ML, Swartz AM, Strath SJ, O’Brien WL, Bassett DR, Schmitz KH, Emplaincourt PO (2000). Compendium of physical activities: an update of activity codes and MET intensities. Med Sci Sports Exerc.

[20] Expert Panel on Detection E (2001). Executive summary of the third report of the National Cholesterol Education Program (NCEP) expert panel on detection, evaluation, and treatment of high blood cholesterol in adults (adult treatment panel III). JAMA.

[21] Ferreira P, Le DC, Zincir-Heywood N. Exploring feature normalization and temporal information for machine learning based insider threat detection. In: 2019 15th International Conference on Network and Service Management (CNSM). IEEE; 2019. p. 1–7.

[22] Chawla NV, Bowyer KW, Hall LO, Kegelmeyer WP (2002). SMOTE: synthetic minority over-sampling technique. J Artif Intell Res.

[23] Pranto AS, Paul MK. Performance Analysis of Ensemble Based Approaches to Mitigate Class Imbalance Problem after Applying Normalization. In: 2021 International Conference on Automation, Control and Mechatronics for Industry 40 (ACMI). IEEE; 2021. p. 1–5.

[24] Cutler A, Cutler DR, Stevens JR. Random forests. In: Ensemble machine learning. Springer; 2012. p. 157–175.

[25] Nilsson PM, Tuomilehto J, Rydén L (2019). The metabolic syndrome—what is it and how should it be managed?. Eur J Prev Cardiol.

[26] Rajkomar A, Dean J, Kohane I (2019). Machine learning in medicine. N Engl J Med.

[27] Pucci G, Alcidi R, Tap L, Battista F, Mattace-Raso F, Schillaci G (2017). Sex-and gender-related prevalence, cardiovascular risk and therapeutic approach in metabolic syndrome: a review of the literature. Pharmacol Res.

[28] Rigatti SJ (2017). Random forest. J Insur Med.

[29] Mohammed R, Rawashdeh J, Abdullah M: Machine learning with oversampling and undersampling techniques: overview study and experimental results. In: 2020 11th international conference on information and communication systems (ICICS). IEEE; 2020. p. 243–248.

[30] Choe EK, Rhee H, Lee S, Shin E, Oh SW, Lee JE, Choi SH (2018). Metabolic syndrome prediction using machine learning models with genetic and clinical information from a nonobese healthy population. Genomics Inform.

